# A frequency domain approach for parameter identification in multibody dynamics

**DOI:** 10.1007/s11044-017-9596-1

**Published:** 2017-11-03

**Authors:** Stefan Oberpeilsteiner, Thomas Lauss, Wolfgang Steiner, Karin Nachbagauer

**Affiliations:** 1 Faculty of Engineering and Environmental Sciences, University of Applied Sciences Upper Austria, Stelzhamerstrasse 23, 4600 Wels, Austria; 2 Institute of Mechanics and Mechatronics, Vienna University of Technology, Getreidemarkt 9/325, 1060 Wien, Austria

**Keywords:** Parameter identification, Frequency domain, Multibody dynamics, Adjoint system, Optimization, Fourieranalysis, Window functions, Engine orders, Order analysis

## Abstract

The adjoint method shows an efficient way to incorporate inverse dynamics to engineering multibody applications, as, e.g., parameter identification. In case of the identification of parameters in oscillating multibody systems, a combination of Fourier analysis and the adjoint method is an obvious and promising approach. The present paper shows the adjoint method including adjoint Fourier coefficients for the parameter identification of the amplitude response of oscillations. Two examples show the potential and efficiency of the proposed method in multibody dynamics.

## Introduction

Applications of the adjoint method to solve a variety of optimization problems in engineering sciences are widespread. Much attention to this approach has been paid recently in the context of multibody systems (see, e.g., [1–7]) in the field of optimal control, sensitivity analysis, and parameter identification. In [8], the adjoint method is seen as a special case of linear duality, which dramatically improves the efficiency of the computation only solving the dual problem. The basic idea of the adjoint method, e.g., as presented in Nachbagauer et al. [7], is the enhancement of the cost function by the system equations of motion including specific system parameters or controls to identify. By including the system equations of motion in the cost function, adjoint variables have to be introduced, leading to the dual problem when solving for these adjoint variables. Minimizing the cost function leads to a classical optimization problem identifying unknown parameters of the system, as, e.g., the mass or inertia parameters of a body or stiffness and damping parameters of a spring-damper force involved. These prescribed examples lead to an identification of parameters in time domain. Various applications of the adjoint method in multibody dynamics for optimal control problems and parameter identifications in time domain can be found in recent works, e.g., in [7]. Adjoint sensitivities have been used in a penalty formulation in time domain for a full vehicle model in [9].

Moreover, from the experiment point of view, the frequency domain plays a key role when analyzing complex multibody systems. Very often only frequency ranges can be investigated in detail. Too low frequencies may not be measured, e.g., with acceleration sensors, and too high frequencies are mainly caused by measuring noise. Identification in the time domain would lead to some kind of best-fit solution. Hence, the goal of the identification is in general to fit a special frequency range. In [10], a system identification for vehicle dynamic applications has been presented based on impulse–momentum equations using a transfer function written as a frequency response function in order to take into account low and high frequency ranges. Spectral element techniques for parameter identification can also be found in the field of layered media in structural dynamics [11]. Therein, the characteristic function of the system, combining the response and impulse force function of the system, is represented in the frequency domain. The transfer function which characterizes the system in the frequency domain is then given as a Fourier transformation. The wavelet transform is used in [12] as a time–frequency representation for the determination of modal parameters of a vibrating system. Therein, natural frequencies, damping ratios, and mode shapes are estimated in the time domain from output data only. A wavelet-based approach for parameter identification is as well presented in [13]. Systems with cubic nonlinearities and systems undergoing both continuous and stick/slip motion have been addressed therein.

The latter mentioned works emphasize the importance of the spectral analysis of the system in order to understand the behavior of the system and consequently be capable of efficient parameter identification. The present paper shows a method for parameter identification in complex multibody systems in the frequency domain. A combination of the adjoint method and classical Fourier analysis for the identification of the amplitude response is presented herein as a novel approach and is applied to engineering problems.

## Problem definition: cost function in terms of Fourier coefficients

Let us consider the system equations of motion in first order form

1 $$ \begin{aligned} \dot{\mathbf{x}}(t) &= \mathbf{f}( \mathbf{x}, \mathbf{p}, t), \quad \mathbf{x}(0) = \mathbf{x}_{0},\\ \mathbf{y}(t) &= \mathbf{g}(\mathbf{x}), \end{aligned} $$

where $\mathbf{p}$ may describe the unknown parameters of the system. For the sake of simplicity, we assume the system has only one output which depends on the states $y(t) := g(\mathbf{x})$. By applying classical Fourier analysis, $y(t)$ can be approximated by

2 $$\begin{aligned} y(t) \approx\frac{1}{2}A_{0} + \sum _{k=1}^{N} \bigl( A_{k}\cos ( \omega_{k} t) + B_{k}\sin(\omega_{k} t) \bigr) , \end{aligned}$$

in which $\omega_{k}$ represents the angular frequency of the $k$th harmonics, each of which is assigned to the appropriate value of its amplitude $\sqrt{A_{k}^{2}+B_{k}^{2}}$. The corresponding Fourier coefficients $A_{k}$ and $B_{k}$ are defined by

3 $$\begin{aligned} A_{k} =& \frac{2}{T} \int_{0}^{T}{y(t)\cos(\omega_{k} t)}\, \mathrm {d}t, \end{aligned}$$

4 $$\begin{aligned} B_{k} =& \frac{2}{T} \int_{0}^{T}{y(t)\sin(\omega_{k} t)}\, \mathrm{d}t. \end{aligned}$$

For example, it might be of interest to determine a set of parameters $\mathbf{p}$ in such a way that the measured amplitudes $\sqrt{\bar{A}_{k}^{2}+\bar{B}_{k}^{2}}$, $k=1,\ldots,N$ of the $k$th harmonics are best approximated by the amplitudes of the system. Therefore, the goal is to find $\mathbf{p}$ such that an error function of the form

5 $$ J = \frac{1}{4}\sum_{k=1}^{N} \bigl[ A_{k}^{2} + B_{k}^{2} - \bigl( \bar{A}_{k}^{2} + \bar{B}_{k}^{2} \bigr) \bigr] ^{2} $$

is minimized. However, the problem may as well be specified in the so-called Mayer form [14]. For that purpose, the Fourier coefficients are introduced by the differential equations

6 $$\begin{aligned} \dot{a}_{k}(t) =& \frac{2}{T} y(t)\cos( \omega_{k} t) = \frac{2}{T} g(\mathbf{x})\cos( \omega_{k} t), \end{aligned}$$

7 $$\begin{aligned} \dot{b}_{k}(t) =& \frac{2}{T} y(t)\sin(\omega_{k} t) = \frac{2}{T} g(\mathbf{x})\sin( \omega_{k} t), \end{aligned}$$

with the corresponding initial conditions $a_{k}(0) = b_{k}(0) = 0$, yielding $A_{k} = a_{k}(T)$ and $B_{k} = b_{k}(T)$. Hence the cost function is considered as a function of the final values of $a_{k}$ and $b_{k}$, i.e., $J = J(A_{k},B_{k})$.

## The adjoint gradient computation

Following the basic idea presented in Nachbagauer et al. [7], the adjoint method is applied to the cost function in Eq. (5). In a first step, the cost function is enhanced by the system equations in Eqs. (1), (6), and (7), leading to

8 $$\begin{aligned} \overline{J} &= J(A_{k},B_{k}) + \int_{0}^{T} \boldsymbol{\xi }^{\mathsf{T}} \bigl( \mathbf{f}(\mathbf{x}, \mathbf{p}, t) - \dot {\mathbf{x}} \bigr) \, \mathrm{d}t \\ &\quad{}+ \sum_{k=1}^{N} \int_{0}^{T} \alpha_{k} \biggl( \frac{2}{T} g(\mathbf{x})\cos(\omega_{k} t) - \dot{a}_{k}(t) \biggr) \, \mathrm {d}t \\ &\quad{}+ \sum_{k=1}^{N} \int_{0}^{T} \beta_{k} \biggl( \frac{2}{T} g(\mathbf {x})\sin(\omega_{k} t) - \dot{b}_{k}(t) \biggr) \, \mathrm{d}t. \end{aligned}$$

Herein $\boldsymbol{\xi}$ represents the vector of adjoint variables corresponding to the state vector. Moreover, $\alpha_{k}$ and $\beta _{k}$ with $k=1,\ldots,N$ are the adjoints related to the Fourier coefficients. Note that $\boldsymbol{\xi}(t)$, $\alpha_{k}(t)$, and $\beta_{k}(t)$ can be arbitrary time functions at this point, since $\overline{J} = J$, if Eqs. (1), (6), and (7) are satisfied. Let us now consider a forward solution $\mathbf{x}(t)$ of the system equations in Eq. (1) for a set of parameters $\mathbf{p}$. A variation $\delta \mathbf{p}$ will result in the variations $\delta \mathbf{x}(t)$, $\delta a_{k}$ and $\delta b_{k}$, respectively, and moreover, in a variation $\delta \bar{J}$. Up to a first order approximation, $\delta\overline{J}$ is given by

9 $$\begin{aligned} \delta\overline{J} &= \sum _{k=1}^{N} \frac{\partial J}{\partial A_{k}} \delta A_{k} + \sum_{k=1}^{N} \frac{\partial J}{\partial B_{k}} \delta B_{k} + \int_{0}^{T} \boldsymbol{\xi}^{\mathsf{T}} ( \mathbf {f}_{\mathbf{x}} \delta\mathbf{x}+ \mathbf{f}_{\mathbf{p}}\delta \mathbf{p}- \delta\dot{\mathbf{x}} ) \, \mathrm{d}t \\ &\quad{}+ \sum_{k=1}^{N} \int_{0}^{T} \biggl( \frac{2}{T} \alpha_{k} g_{\mathbf{x}} \delta\mathbf{x}\cos(\omega_{k} t) - \alpha_{k} \delta\dot{a}_{k} \biggr) \, \mathrm{d}t \\ &\quad{}+ \sum_{k=1}^{N} \int_{0}^{T} \biggl( \frac{2}{T} \beta_{k} g_{\mathbf{x}} \delta\mathbf{x}\sin(\omega_{k} t) - \beta_{k} \delta\dot{b}_{k} \biggr) \, \mathrm{d}t. \end{aligned}$$

After applying integration by parts to the terms including $\delta\dot {\mathbf{x}}$, $\delta\dot{a}_{k}(t)$, and $\delta\dot{b}_{k}(t)$, the variation can be written in the form

10 $$\begin{aligned} \delta\overline{J} &= \sum _{k=1}^{N} \frac{\partial J}{\partial A_{k}} \delta A_{k} + \sum_{k=1}^{N} \frac{\partial J}{\partial B_{k}} \delta B_{k} \\ &\quad{}+ \int_{0}^{T} \boldsymbol{\xi}^{\mathsf{T}} ( \mathbf {f}_{\mathbf{x}} \delta\mathbf{x}+ \mathbf{f}_{\mathbf{p}}\delta \mathbf{p} ) \,dt + \int_{0}^{T} \dot{\boldsymbol{\xi}}^{\mathsf {T}} \delta\mathbf{x}\, \mathrm{d}t- \boldsymbol{\xi}^{\mathsf{T}} (T) \delta \mathbf{x}(T) \\ &\quad{}+ \sum_{k=1}^{N} \biggl( \int_{0}^{T} \frac{2}{T} \alpha_{k} g_{\mathbf{x}} \delta\mathbf{x}\cos(\omega_{k} t) \, \mathrm{d}t+ \int_{0}^{T} \dot{\alpha}_{k} \delta a_{k} \, \mathrm{d}t- \alpha _{k}(T) \delta A_{k} \biggr) \\ &\quad{}+ \sum_{k=1}^{N} \biggl( \int_{0}^{T} \frac{2}{T} \beta_{k} g_{\mathbf{x}} \delta\mathbf{x}\sin(\omega_{k} t) \, \mathrm{d}t+ \int_{0}^{T} \dot{\beta}_{k} \delta b_{k} \, \mathrm{d}t- \beta _{k}(T) \delta B_{k} \biggr) , \end{aligned}$$

where the initial conditions $\delta\mathbf{x}(0) = \mathbf{0}$, $\delta a_{k}(0) = \delta b_{k}(0) = 0$ and end conditions $\delta a_{k}(T) = \delta A_{k}$, $\delta b_{k}(T) = \delta B_{k}$ are used. The computation of $\delta\mathbf{x}$, $\delta a_{k}$, and $\delta b_{k}$ can be circumvented, if the factors multiplied vanish. First, the terms including $\delta a_{k}$ and $\delta b_{k}$ disappear, if $\dot{\alpha}_{k} = \dot{\beta}_{k}=0$, i.e., if $\alpha_{k}=\text{const.}=\alpha_{k}(T)$ and $\beta_{k}=\text{const.}=\beta_{k}(T)$.

Second, the terms including $\delta\mathbf{x}$ vanish, if the adjoints $\boldsymbol{\xi}$ are defined by

11 $$ \dot{\boldsymbol{\xi}} = -\mathbf{f}_{\mathbf{x}}^{\mathsf {T}} \boldsymbol{\xi}- \frac{2}{T}g_{\mathbf{x}}^{\mathsf{T}} \sum _{k=1}^{N} \bigl( \alpha_{k} \cos( \omega_{k} t) + \beta_{k} \sin (\omega_{k} t) \bigr). $$

The boundary conditions are chosen such that $\boldsymbol{\xi}(T) = \mathbf{0}$ in order to eliminate the coefficients of $\delta\mathbf {x}(T)$ in Eq. (10). Finally, the terms multiplied with $\delta A_{k}$ and $\delta B_{k}$ can be eliminated by defining $\alpha_{k}(T)$ and $\beta_{k}(T)$ from

12 $$\begin{aligned} \alpha_{k} =& \alpha_{k}(T) = \frac{\partial J}{\partial A_{k}}, \end{aligned}$$

13 $$\begin{aligned} \beta_{k} =&\beta_{k}(T) = \frac{\partial J}{\partial B_{k}}. \end{aligned}$$

With $\mathbf{x}(t)$ from the forward solution of the system equations in Eq. (1) and the backward solution for $\boldsymbol{\xi}(t)$ of the adjoint system in Eq. (11), the variation of $\overline{J}$ is readily given by

14 $$ \delta\overline{J} = \int_{0}^{T} \boldsymbol{\xi}^{\mathsf{T}} \mathbf{f}_{\mathbf{p}}\delta\mathbf{p}\, \mathrm{d}t= \biggl( \int _{0}^{T} \boldsymbol{\xi}^{\mathsf{T}} \mathbf{f}_{\mathbf{p}}\, \mathrm{d}t \biggr) \delta\mathbf{p}, $$

which is in accordance with the variation of the original cost function $\delta J$. Hence, the gradient of $J$ reads

15 $$ \frac{\partial J}{\partial\mathbf{p}} = \int_{0}^{T} \boldsymbol{\xi }^{\mathsf{T}} \mathbf{f}_{\mathbf{p}}\,\mathrm{d}t. $$

## Application to multibody systems

In most cases, multibody systems are described by a system of differential algebraic equations (DAE)

16 $$ \begin{aligned} \mathbf{M}(\mathbf{q}) \ddot{\mathbf{q}} + \mathbf{C}_{\mathbf {q}}^{\mathsf{T}}(\mathbf{q})\boldsymbol{\lambda}&= \mathbf{f}(\mathbf {q},\dot{\mathbf{q}},\mathbf{p},t), \\ \mathbf{C}(\mathbf{q}) &= \textbf{0}, \end{aligned} $$

in which $\mathbf{q}$ denotes the vector of redundant generalized coordinates, $\mathbf{M}$ the symmetric mass matrix, and $\mathbf{f}$ the vector of generalized and gyroscopic forces. Due to the algebraic constraints $\mathbf{C}(\mathbf{q}) = \textbf{0}$, the equations of motion are extended by constraint forces of the form $\mathbf{C}_{\mathbf{q}}^{\mathsf{T}}\boldsymbol{\lambda}$, where $\mathbf{C}_{\mathbf{q}}$ represents the constraint Jacobian and the vector $\boldsymbol{\lambda}$ includes the Lagrange multipliers. Moreover, $\mathbf{p}$ is the vector of system parameters.

Using the additional variables $\mathbf{v}=\dot{\mathbf{q}}$, the equations of motion can be reformulated as the following first order system of equations:

17 $$ \begin{aligned} \dot{\mathbf{q}} &= \mathbf{v}, \\ \mathbf{M}\dot{\mathbf{v}} + \mathbf{C}_{\mathbf{q}}^{\mathsf {T}}\boldsymbol{ \lambda}&= \mathbf{f}(\mathbf{q},\mathbf{v},\mathbf {p},t), \\ \mathbf{C}(\mathbf{q}) &= \textbf{0}. \end{aligned} $$

In this setting, the system output depending on $\mathbf{q}$ and $\mathbf{v}$ is denoted again by $y(t)=g(\mathbf{q},\mathbf{v})$.

The differential equations for the Fourier coefficients can be formulated analogously to Eqs. (6) and (7) by

18 $$\begin{aligned} \dot{a}_{k}(t) &= \frac{2}{T} g(\mathbf{q},\mathbf{v}) \cos( \omega _{k} t) \quad\text{with}\ a_{k}(0)=0, \end{aligned}$$

19 $$\begin{aligned} \dot{b}_{k}(t) &= \frac{2}{T} g(\mathbf{q},\mathbf{v}) \sin( \omega _{k} t) \quad\text{with}\ b_{k}(0)=0. \end{aligned}$$

Again, the goal is to find a set of parameters $\mathbf{p}$ such that the cost function in Eq. (5) is minimized. The enhancement of the cost function by the system equations in first order form in Eq. (17) and by the additional differential equations for the Fourier coefficients in Eqs. (18) and (19) leads to

20 $$\begin{aligned} \begin{aligned}[b] \overline{J} &= J(A_{k},B_{k}) + \int_{0}^{T} \bigl\{ \boldsymbol{\xi }^{\mathsf{T}} ( \dot{\mathbf{q}} - \mathbf{v} ) + \boldsymbol {\zeta}^{\mathsf{T}} \bigl( \mathbf{M}\dot{\mathbf{v}} - \mathbf {f}(\mathbf{q},\mathbf{v},\mathbf{p},t) + \mathbf{C}_{\mathbf {q}}^{\mathsf{T}}\boldsymbol{\lambda} \bigr) + \boldsymbol{ \mu }^{\mathsf{T}} \mathbf{C}(\mathbf{q}) \bigr\} \, \mathrm{d}t \\ &\quad{}+\sum_{k=1}^{N} \int_{0}^{T} \alpha_{k} \biggl( \frac{2}{T} g(\mathbf {q},\mathbf{v})\cos(\omega_{k} t) - \dot{a}_{k}(t) \biggr) \,\mathrm {d}t \\ &\quad{}+\sum_{k=1}^{N} \int_{0}^{T} \beta_{k} \biggl( \frac{2}{T} g(\mathbf {q},\mathbf{v})\sin(\omega_{k} t) - \dot{b}_{k}(t) \biggr) \,\mathrm{d}t,\end{aligned} \end{aligned}$$

in which $\boldsymbol{\xi}$ and $\boldsymbol{\zeta}$ represent the adjoint variables corresponding to the multibody system, $\boldsymbol {\mu}$ pertains to the constraint equation, and $\alpha_{k}$ and $\beta_{k}$ with $k=1,\ldots,N$ are the adjoints corresponding to the Fourier coefficients $a_{k}$ and $b_{k}$, respectively. At this point, the variables $\boldsymbol{\xi}(t)$, $\boldsymbol{\zeta}(t)$, $\boldsymbol{\mu}(t)$, $\alpha_{k}$ and $\beta_{k}$ may be chosen arbitrarily. The variation of the function $\overline{J}$ is given by

21 $$\begin{aligned} \delta\overline{J} &= \sum _{k=1}^{N} \frac{\partial J}{\partial A_{k}} \delta A_{k} + \sum_{k=1}^{N} \frac{\partial J}{\partial B_{k}} \delta B_{k} \\ &\quad{}+ \int_{0}^{T} \bigl\{ \boldsymbol{\xi}^{\mathsf{T}} (\delta\dot {\mathbf{q}} - \delta\mathbf{v}) + \boldsymbol{\mu}^{\mathsf {T}} \mathbf{C}_{\mathbf{q}}\delta\mathbf{q} \\ &\quad{}+ \boldsymbol{\zeta}^{\mathsf{T}} \bigl[ ( \mathbf{M}\dot{\mathbf {v}} ) _{\mathbf{q}}\delta\mathbf{q}+ \mathbf{M}\delta\dot {\mathbf{v}} - \mathbf{f}_{\mathbf{q}}\delta\mathbf{q}- \mathbf {f}_{\mathbf{v}}\delta \mathbf{v}- \mathbf{f}_{\mathbf{p}}\delta\mathbf {p}+ \bigl( \mathbf{C}_{\mathbf{q}}^{\mathsf{T}}\boldsymbol{\lambda } \bigr) _{\mathbf{q}}\delta\mathbf{q}+ \mathbf{C}_{\mathbf {q}}^{\mathsf{T}}\delta \boldsymbol{\lambda} \bigr] \bigr\} \, \mathrm {d}t \\ &\quad{}+ \sum_{k=1}^{N} \int_{0}^{T} \biggl\{ \frac{2}{T} \alpha_{k} \bigl( g_{\mathbf{q}} \cos(\omega_{k} t) \delta \mathbf{q}+ g_{\mathbf{v}} \cos(\omega_{k} t) \delta\mathbf{v} \bigr) - \alpha_{k} \delta\dot {a}_{k} \biggr\} \, \mathrm{d}t \\ &\quad{}+ \sum_{k=1}^{N} \int_{0}^{T} \biggl\{ \frac{2}{T} \beta_{k} \bigl( g_{\mathbf{q}} \sin(\omega_{k} t) \delta \mathbf{q}+ g_{\mathbf{v}} \sin(\omega_{k} t) \delta\mathbf{v} \bigr) - \beta_{k} \delta\dot {b}_{k} \biggr\} \, \mathrm{d}t. \end{aligned}$$

Integrating by parts of the terms with $\delta\dot{\mathbf{q}}$, $\delta\dot{\mathbf{v}}$, $\delta\dot{a}_{k}$, $\delta\dot{b}_{k}$ and assuming that $\delta\mathbf{q}(0) = 0$, $\delta\mathbf{v}(0) = 0$, $\delta a_{k}(0) = 0$ and $\delta b_{k}(0) = 0$, as a consequence of prescribed initial conditions for $q$, $v$, $a_{k}$ and $b_{k}$, yields

22 $$\begin{aligned} \begin{aligned}[b] \delta\overline{J} &= \sum _{k=1}^{N} \frac{\partial J}{\partial A_{k}} \delta A_{k} + \sum_{k=1}^{N} \frac{\partial J}{\partial B_{k}} \delta B_{k} \\ &\quad{}+ \int_{0}^{T} \biggl\{ -\dot{\boldsymbol{ \xi}}^{\mathsf{T}} \delta \mathbf{q}- \boldsymbol{\xi}^{\mathsf{T}}\delta \mathbf{v}+ \boldsymbol {\zeta}^{\mathsf{T}} ( \mathbf{M}\dot{\mathbf{v}} ) _{\mathbf {q}}\delta\mathbf{q}-\frac{d}{\mathrm{d}t}\bigl( \boldsymbol{\zeta }^{\mathsf{T}}\mathbf{M}\bigr)\delta\mathbf{v}- \boldsymbol{\zeta}^{\mathsf {T}} \mathbf{f}_{\mathbf{q}}\delta\mathbf{q}- \boldsymbol{\zeta }^{\mathsf{T}} \mathbf{f}_{\mathbf{v}}\delta\mathbf{v} \\ &\quad{}- \boldsymbol{\zeta}^{\mathsf{T}}\mathbf{f}_{\mathbf{p}}\delta\mathbf {p}+ \boldsymbol{\zeta}^{\mathsf{T}} \bigl( \mathbf{C}_{\mathbf {q}}^{\mathsf{T}} \boldsymbol{\lambda} \bigr) _{\mathbf{q}}\delta \mathbf{q}+ \boldsymbol{ \zeta}^{\mathsf{T}}\mathbf{C}_{\mathbf {q}}^{\mathsf{T}}\delta\boldsymbol{ \lambda}+ \boldsymbol{\mu }^{\mathsf{T}}\mathbf{C}_{\mathbf{q}}\delta\mathbf{q} \biggr\} \,\mathrm {d}t \\ &\quad{}+ \sum_{k=1}^{N} \int_{0}^{T} \biggl\{ \frac{2}{T} \alpha_{k} \bigl( g_{\mathbf{q}} \cos(\omega_{k} t) \delta \mathbf{q}+ g_{\mathbf {v}}\cos(\omega_{k} t) \delta\mathbf{v} \bigr) + \dot{\alpha}_{k} \delta a_{k} \biggr\} \, \mathrm{d}t \\ &\quad{}+ \sum_{k=1}^{N} \int_{0}^{T} \biggl\{ \frac{2}{T} \beta_{k} \bigl( g_{\mathbf{q}} \sin(\omega_{k} t) \delta \mathbf{q}+ g_{\mathbf {v}}\sin(\omega_{k} t) \delta\mathbf{v} \bigr) + \dot{\beta}_{k} \delta b_{k} \biggr\} \, \mathrm{d}t \\ &\quad{}+ \bigl( \boldsymbol{\xi}^{\mathsf{T}}\delta\mathbf{q}+ \boldsymbol { \zeta}^{\mathsf{T}}\mathbf{M}\delta\mathbf{v} \bigr) |_{t=T} - \sum _{k=1}^{N} \bigl( \alpha_{k}(T) \delta A_{k} + \beta_{k}(T)\delta B_{k} \bigr). \end{aligned} \end{aligned}$$

The computation of the variations $\delta a_{k}$, $\delta b_{k}$, $\delta\mathbf{q}$, $\delta\mathbf{v}$, and $\delta\boldsymbol {\lambda}$ can be circumvented, if the factors multiplied vanish. In case of the adjoints $\alpha_{k}$ and $\beta_{k}$, constant values $\alpha_{k} = \alpha_{k}(T) = \frac{\partial J}{\partial A_{k}}$ and $\beta_{k} = \beta_{k}(T) = \frac{\partial J}{\partial B_{k}}$ are used to fulfill $\dot{\alpha}_{k} = \dot{\beta}_{k} = 0$. The adjoint variables $\boldsymbol{\xi}(t)$, $\boldsymbol{\zeta}(t)$, and $\boldsymbol{\mu}(t)$ have to be chosen such that the adjoint equations

23 $$ \begin{aligned} \dot{\boldsymbol{\xi}} &= \mathbf{A}\boldsymbol{\zeta}+ \mathbf {C}_{\mathbf{q}}^{\mathsf{T}}\boldsymbol{\mu}+ g_{\mathbf{q}}\mathbf {G}(t), \\ \frac{\mathrm{d}}{\mathrm{d}t}(\mathbf{M}\boldsymbol{\zeta}) &= - \boldsymbol{\xi}- \mathbf{f}_{\mathbf{v}}^{\mathsf{T}}\boldsymbol {\zeta}+ g_{\mathbf{v}} \mathbf{G}(t), \\ \mathbf{C}_{\mathbf{q}}\boldsymbol{\zeta}&= \mathbf{0} \end{aligned} $$

hold. Here the terms $\mathbf{A}= ( \mathbf{M}\dot{\mathbf {v}} ) _{\mathbf{q}}^{\mathsf{T}} - \mathbf{f}_{\mathbf {q}}^{\mathsf{T}} + ( \mathbf{C}_{\mathbf{q}}^{\mathsf {T}}\boldsymbol{\lambda} ) _{\mathbf{q}}^{\mathsf{T}}$ and $\mathbf{G}(t) = \sum_{k=1}^{N} \frac{2}{T} (\alpha_{k} \cos (\omega_{k} t) + \beta_{k} \sin(\omega_{k} t) )$ are used. At this point the adjoints can be chosen arbitrarily at $t=T$. For the sake of simplicity, they are set to zero, $\boldsymbol{\xi}(T) = \boldsymbol{\zeta}(T) = \boldsymbol{\mu}(T) = \mathbf{0}$, in order to eliminate the corresponding boundary terms.

It has to be mentioned here that the symmetry of the mass matrix $\mathbf{M}=\mathbf{M}^{\mathsf{T}}$ has been used. If Eqs. (23) are satisfied, the variation $\delta \overline{J}$ reduces to

24 $$ \delta\overline{J} = \int_{0}^{T} \bigl( - \boldsymbol{\zeta }^{\mathsf{T}}\mathbf{f}_{\mathbf{p}}\delta\mathbf{p} \bigr) \mathrm {d}t= \biggl( \frac{\partial\overline{J}}{\partial\mathbf{p}} \biggr) ^{\mathsf{T}} \delta\mathbf{p} $$

which directly relates the independent variation $\delta\mathbf{p}$ to the variation of the objective function. Hence, we may conclude that the gradient of $\overline{J}$ is given by

25 $$ \nabla\overline{J} = \int_{0}^{T} \bigl( - \boldsymbol{\zeta }^{\mathsf{T}}\mathbf{f}_{\mathbf{p}} \bigr) \, \mathrm{d}t. $$

## Numerical examples

### Cart pendulum system

**Fig. 1:**
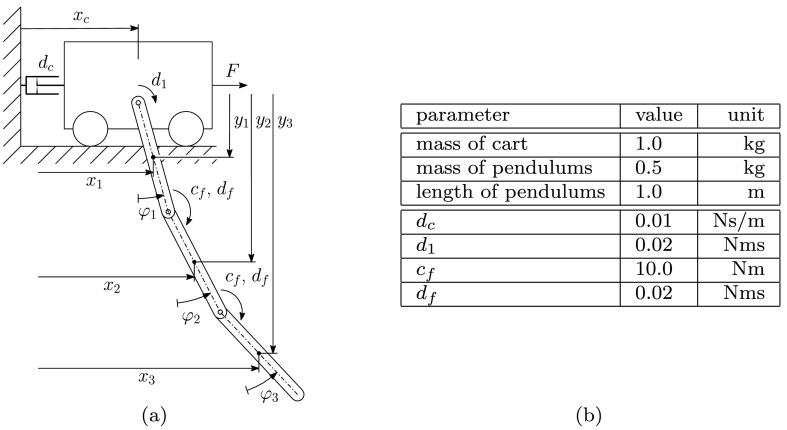
A system consisting of a cart and three rigid pendula is studied, where the parameters of the flexible pendulum are identified for a given excitation $F$. (**a**) Geometric description of the cart–pendulum system. (**b**) Definition of the parameters used for simulating the system

In order to present the performance of the identification method, we study a system of pendula connected to a cart performing a one-dimensional motion. As it can be seen in Fig. 1(a), the pendula are interconnected with rotational springs, and therefore this configuration represents a discretization of a rotating flexible beam. In this example we assume the parameters of the flexible beam, the stiffness $c_{f}$, and damping coefficient $d_{f}$ to be unknown. The parameter $d_{c}$, representing the cart’s friction, remains untouched during the identification process at a prescribed value.

**Fig. 2:**
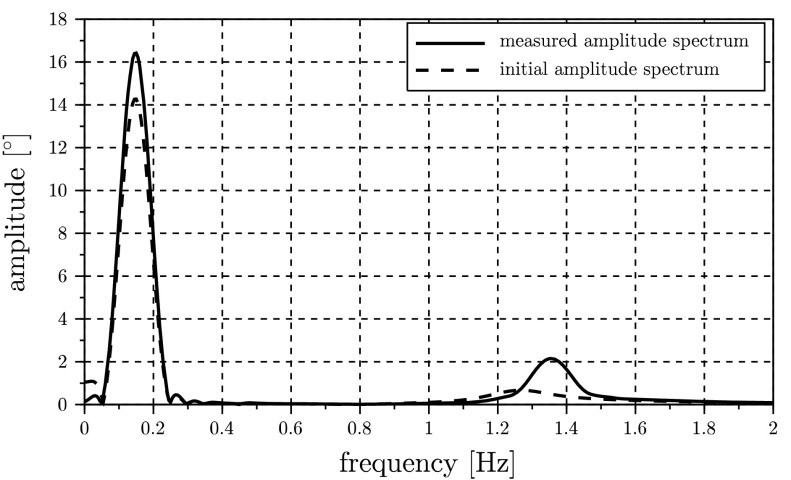
Measured and initial amplitude spectrum of pendulum angle $\varphi_{1}$

The desired spectrum needed for the computation of the cost function $J$ in Eq. (5) is generated by using the angle $\varphi_{1}(t)$ as a system output. For this purpose a numerical simulation utilizing the parameters listed in the table in Fig. 1(b) is performed in order to obtain some kind of virtual measurement. The resulting amplitude spectrum is shown in Fig. 2.

**Fig. 3:**
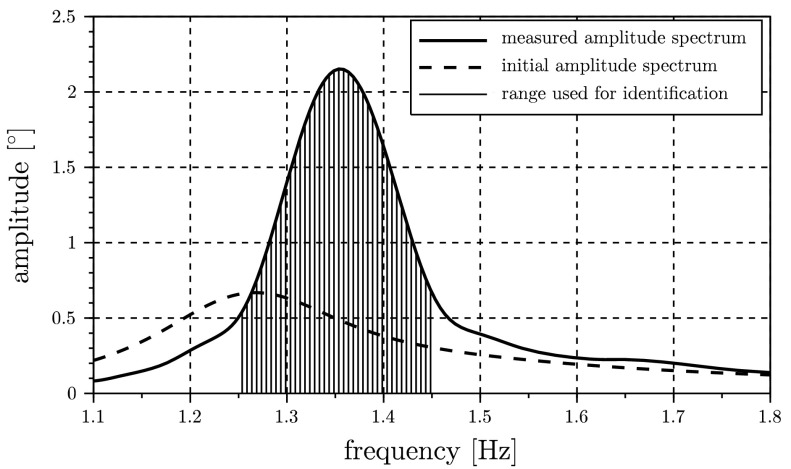
Spectrum of pendulum angle $\varphi_{1}$ used for identification (interval highlighted)

The actual parameter identification is initiated with the parameters $c_{f}=8.5~\text{Nm}$ and $d_{f}=0.15~\text{Nms}$, and therefore the initial spectrum presented in Fig. 3 differs from the desired one. In order to include an additional model uncertainty, the prescribed damping parameter $d_{c}$ is modified to $0.1~\text{Ns/m}$ for the identification of $c_{f}$ and $d_{f}$, whereas the value $d_{c} = 0.01~\text{Ns/m}$ is used for generating the virtual measurement. This allows for pointing out the main advantage of the presented method, which is the possibility to filter data and perform the identification in frequency intervals of interest only. The damping parameter $d_{c}$ mainly affects the amplitude occurring around the first eigenfrequency of the system at about $0.17~\text{Hz}$, which correlates with the rigid body mode, whereas the eigenfrequency of the first bending mode is located in the interval $[1.25,1.45]~\text{Hz}$. Due to this fact, just the amplitudes in this interval are considered as desired spectrum. When thinking about real applications, this consideration may help to perform a parameter identification even though some subsystems are not known to full extent.

**Fig. 4:**
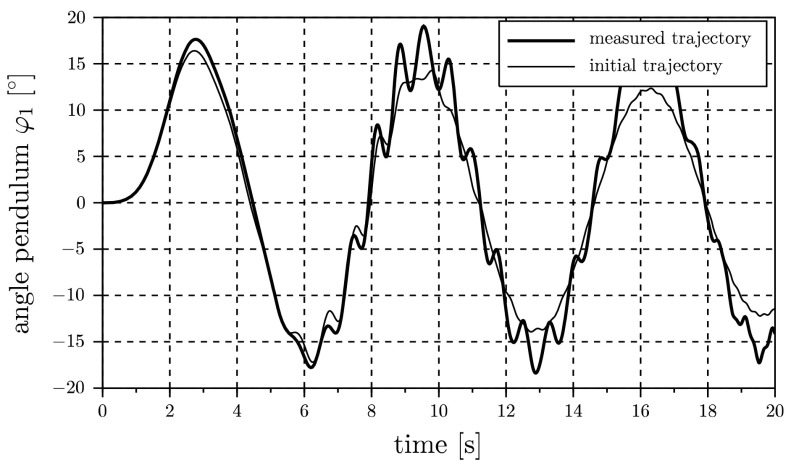
Comparison of pendulum angle $\varphi_{1}$ in the time domain

In order to demonstrate the difference from identification performed in the time domain, in Fig. 4 the pendulum angle $\varphi_{1}$ is plotted for both parameter combinations. Due to the increased damping coefficient $d_{c}$, the amplitude of the initial trajectory differs significantly from the measured one, especially at the end of the experiment. In the time domain there is no opportunity of filtering data, and therefore this approach would lead to incorrect parameters $c_{f}$ and $d_{f}$.

**Fig. 5:**
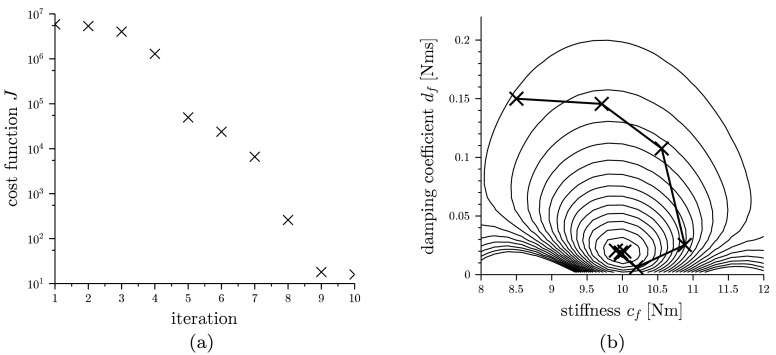
(**a**) Convergence history for optimization of parameters $c_{f}$ and $d_{f}$, (**b**) Contour plot of cost function $J$ for parameters $c_{f}$ and $d_{f}$

By using a quasi-Newton method like the BFGS algorithm for finding a minimum of $J$ and incorporating the computed gradient of Eq. (25), the solution can be found within 10 iterations. In Fig. 5(a) the convergence history for the optimization process is shown. The contour plot in Fig. 5(b) gives an impression of the complex shape of $J(c_{f},d_{f})$ and displays the optimization path taken by the BFGS algorithm. The final parameters gained by using the presented method are $c_{f} = 9.98~\text{Nm}$ and $d_{f} = 0.019~\text{Nms}$ compared to the values used for generating the measure $c_{f} = 10.0~\text{Nm}$ and $d_{f} = 0.02~\text{Nms}$. The slight difference to the expected parameters can be explained by the remaining influence of the damping $d_{c}$ onto the spectrum in the interval investigated, which was different for the model and the (virtual) measurement as explained above.

### Identification of torsional vibration damper (TVD) parameters

**Fig. 6:**
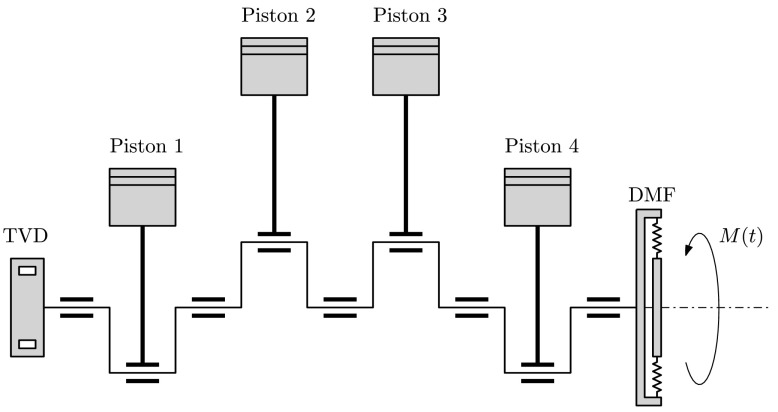
Schematics of a four-cylinder engine

In this section the presented method is applied to a model of a four-cylinder engine, shown in Fig. 6. The detailed model equations are given in Appendix A. The goal is to identify the parameters of the engine’s torsional vibration damper (TVD), which is described by two Maxwell elements. The TVD is installed in order to reduce torsional oscillations of the crankshaft, which show large amplitudes at the 6th engine order.

#### Model structure

##### Crankshaft

**Fig. 7:**
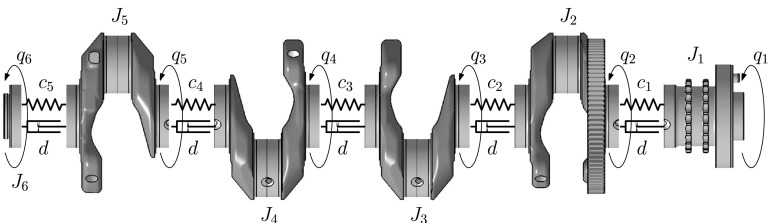
Discretization of the crankshaft

The torsional vibration modes of the crankshaft are crucial for the parameter identification process. Therefore, six lumped masses resulting in six degrees of freedom $q_{1},\dots,q_{6}$ (see Fig. 7) are introduced in order to model the structural flexibility of the crankshaft. The masses are interconnected with linear springs $c_{1},\dots,c_{6}$ and linear damping elements $d_{1},\dots,d_{5}$, respectively. The inertia parameters are given by the moments of inertia $J_{1},\dots,J_{6}$.

##### Conrod

**Fig. 8:**
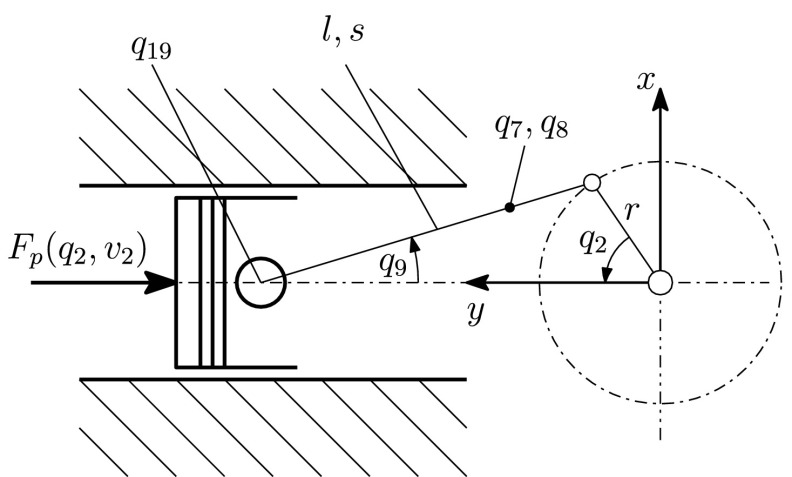
Slider–crank mechanism (for cylinder 1)

For describing the in-plane motion of an engine’s connecting rod (conrod) three degrees of freedom are introduced. According to Fig. 8, two degrees of freedom are used for the translational motion and one for the rotation about the rotation axis. The mass of each conrod is given by $m_{\text{cr}}$ and the moment of inertia by $J_{\text{cr}}$.

##### Piston

In the used model, each piston features only one translational degree of freedom. The mass of each piston is denoted as $m_{\text{p}}$ and the piston’s effective area as $A_{P}$.

##### Dual mass flywheel

The primary side of the dual mass flywheel (DMF) is mounted on the right end of the crankshaft (see Fig. 7). Hence, its moment of inertia is assigned to $q_{1}$. Instead of introducing a degree of freedom for the secondary side of the DMF, the prescribed angle $q_{\text{runup}}(t)$ is used. A nonlinear torsional spring and a linear damping element is used for connecting the primary with the secondary side.

##### Torsional vibration damper

**Fig. 9:**
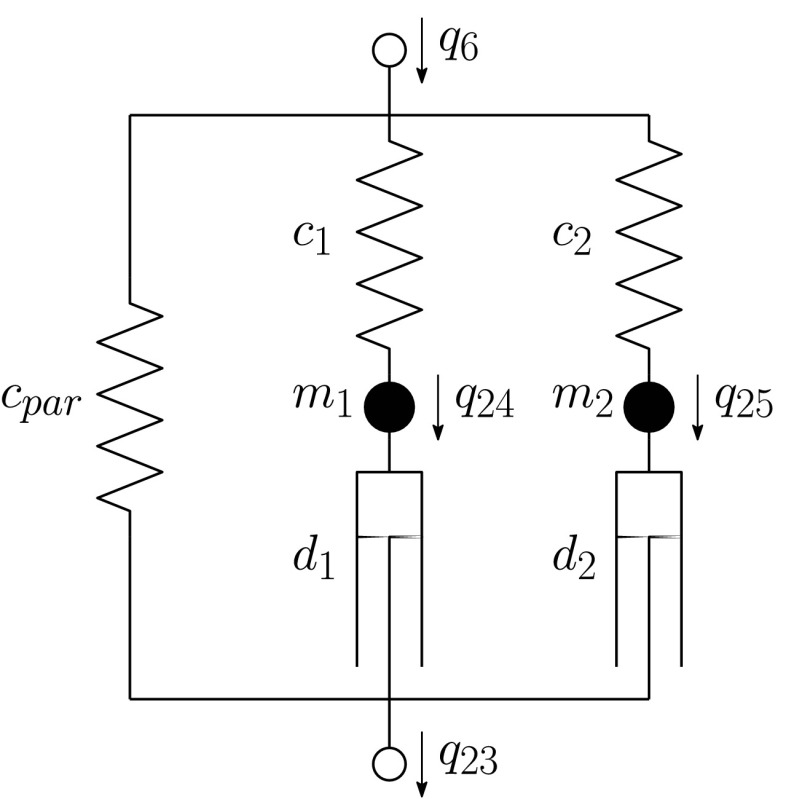
Model of the torsional vibration damper

The torsional vibration damper (TVD) is installed for reducing the internal torsional vibrations of the crankshaft. Within the case of the TVD, a flywheel ring is gliding in a viscous fluid. Usually, the mathematical model of the TVD is approximated by Maxwell elements. In this contribution, two Maxwell elements and one parallel spring are used. In Fig. 9 a schematic description of the model and the degrees of freedom used are presented. The accuracy of the parameters $c^{*}_{1}$, $c^{*}_{2}$, $d^{*}_{1}$, $d^{*}_{2}$, and $c^{*}_{\text{par}}$ supplied by the manufacturer is not satisfactory, and therefore the values of the four parameters $c^{*}_{1}$, $c^{*}_{2}$, $d^{*}_{1}$, and $d^{*}_{2}$ are adjusted by using parameter identification in the frequency domain. The masses $m^{*}_{1}$ and $m^{*}_{2}$ are set to zero.

##### Pulley wheel

The pulley wheel used for driving additional aggregates introduces another degree of freedom ($q_{26}$), which is connected to the TVD hub using a linear spring/damper with parameters $c_{PW}$ and $d_{PW}$.

##### Cylinder pressures

The cylinder pressure is given by a two-dimensional map depending on the rotational speed and the crankshaft angle. The pressure is applied on each piston in accordance with the firing order.

##### Run-up of the engine

**Fig. 10:**
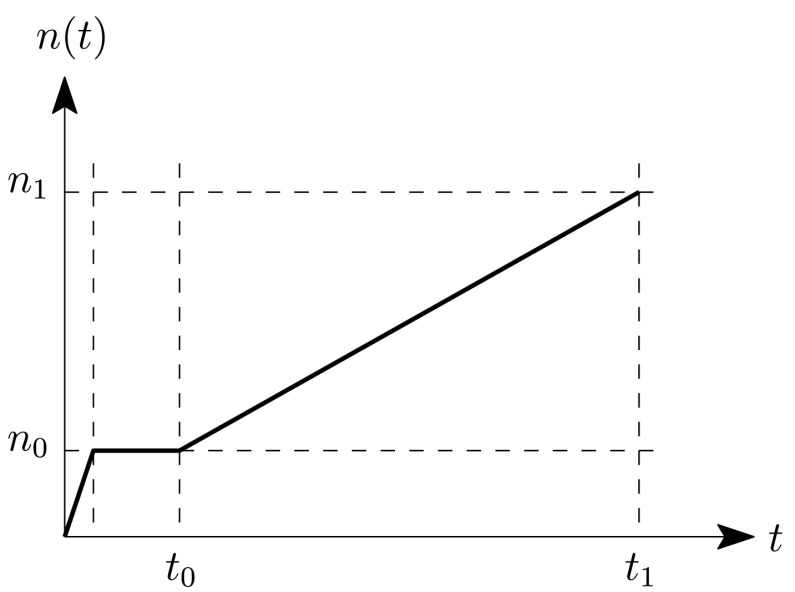
Rotational speed of the engine run-up

In order to simulate the run-up performed on the real test bench, the rotational speed of the secondary side of the DMF is increased up to the final rotational speed. The ramp used for the simulation is given in Fig. 10. The run-up to the final rotational speed itself is done within the time interval $[t_{0}, t_{1}]$.

#### Results of the parameter identification

In this section the identification of four parameters $c^{*}_{1}$, $c^{*}_{2}$, $d^{*}_{1}$, and $d^{*}_{2}$ of the torsional vibration damper is presented. The main purpose of the TVD is to reduce torsional vibrations of the flexible crankshaft caused by the periodic and dynamic loads (e.g., cylinder pressures). Hence, the twist angle of the crankshaft $y(t) = q_{6}(t) - q_{1}(t)$ is chosen to be transformed into the frequency domain. As the measurements performed on a test bench commonly result in spectra for different engine orders, they are used in this investigation, too. Basically, an engine order relates the Fourier coefficients with the rotating frequencies of the engine’s crankshaft. In case of the four cylinder engine, the amplitude of the 6th order is dominated by the parameter of the torsional vibration damper. In contrast to Eqs. (6) and (7), the differential equations for computing the Fourier coefficients are

$$ \begin{aligned} \dot{a}_{k} &= \frac{2}{t_{f}} \eta(t)y(t)\cos(\omega_{k} t), \\ \dot{b}_{k} &= \frac{2}{t_{f}} \eta(t)y(t)\sin( \omega_{k} t), \end{aligned} $$

where $\eta(t)$ represents a window function and $\omega_{k}$ the $k$th frequency of interest. It has been shown that the Hanning window (or Von-Hann window) given by

26 $$ \eta(t) = \frac{g_{\text{c}}}{2} \biggl[ 1-\cos \biggl( \frac{\pi }{t_{\text{u}}-t_{\text{l}}} (t-t_{\text{l}}) \biggr) \biggr] $$

is a good choice for filtering the system output. The upper time limit $t_{\text{u}}$ and the lower time limit $t_{\text{l}}$ determine the borders of the window function. Note that an amplitude correction factor $g_{\text{c}}=2$ is required (for further details see Appendix B).

**Fig. 11:**
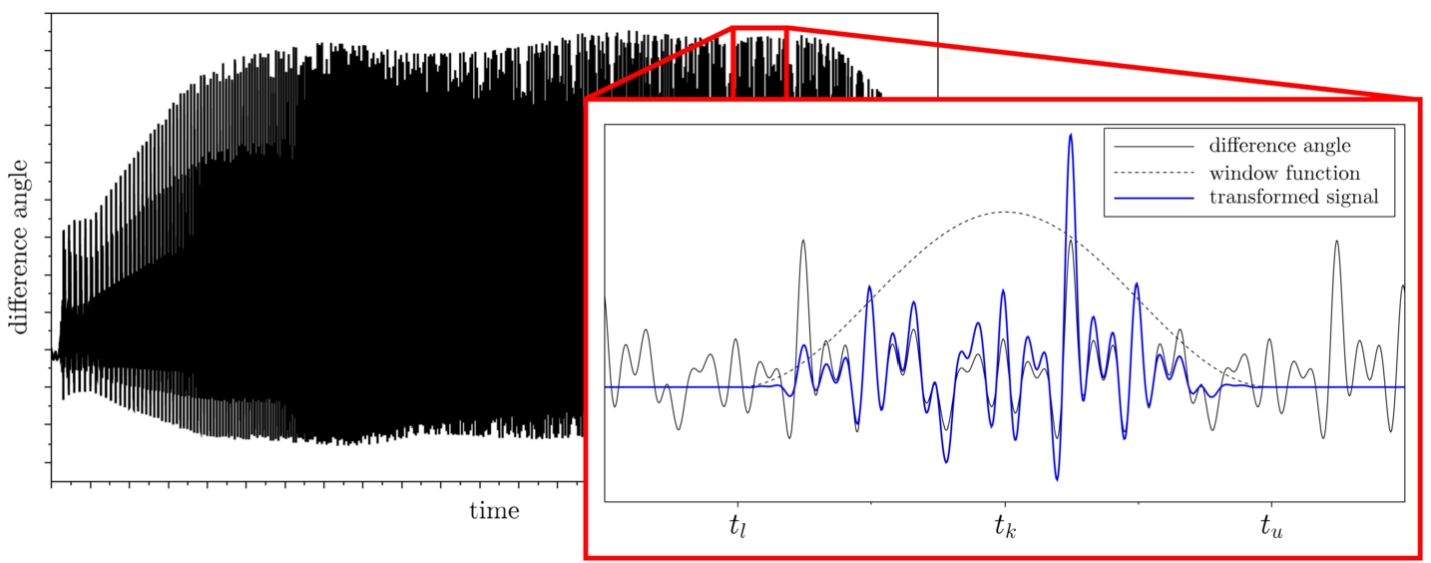
Window function and twist angle

In Fig. 11 the system output $y(t)$ is shown for the entire time interval. Moreover, a small interval $t\in[t_{l},t_{u}]$ of $y(t)$ is depicted in detail. The black line shows the original system output $y(t)$, while the dashed line shows the Hanning window function $\eta(t)$. The blue line is the multiplication of $y(t)$ with $\eta(t)$, which is used for the Fourier transformation. Due to the slowly increasing ramp shown in Fig. 10, assuming a steady state with constant angular velocity is valid. For the rotational speed $n$ the time interval $[t_{l},t_{u}]$ is given by

$$ t_{l} = t_{k} - \frac{2}{n} \quad\text{and} \quad t_{u} = t_{k} + \frac{2}{n} $$

with

$$ t_{k} = \frac{n_{0}\,t_{1}-n_{1}\,t_{0} + n\,(t_{0} - t_{1})}{n_{0}- n_{1}}. $$

Here two periods of the base frequency, which is $2/n$ for a four stroke engine, are contained in the Hanning window (for further details see Appendix B).

**Fig. 12:**
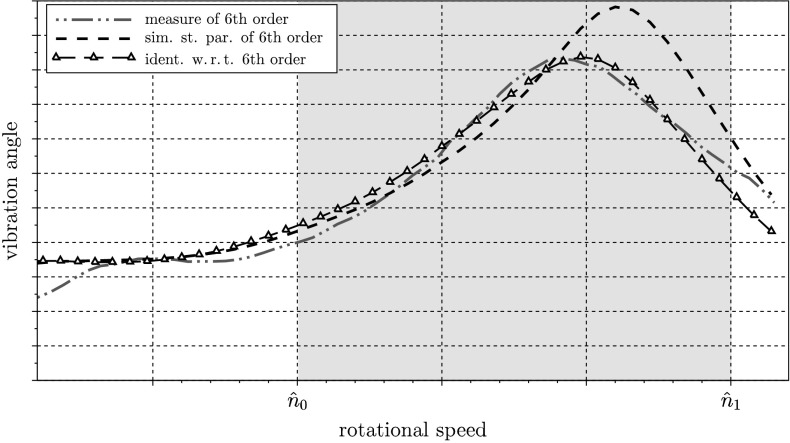
Vibration angle of the 6th engine order

In Fig. 12 the dashed line shows the vibration angle corresponding to the 6th engine order using initial parameters of the TVD. The dotted–dashed line (green) represents the measured vibration angle of the 6th engine order on a test bench. The black line (with the triangle symbol) shows the vibration angle of the 6th engine order with the identified TVD parameters. In the interval $n \in[\hat{n}_{0}, \hat{n}_{1}]$, highlighted in Fig. 12, the deviation between the measured amplitude and the simulated amplitude is included in the cost function of Eq. (5). Hence, a significant improvement can be seen in this range compared to the simulation utilizing the initial parameters of the torsional vibration damper.

**Fig. 13:**
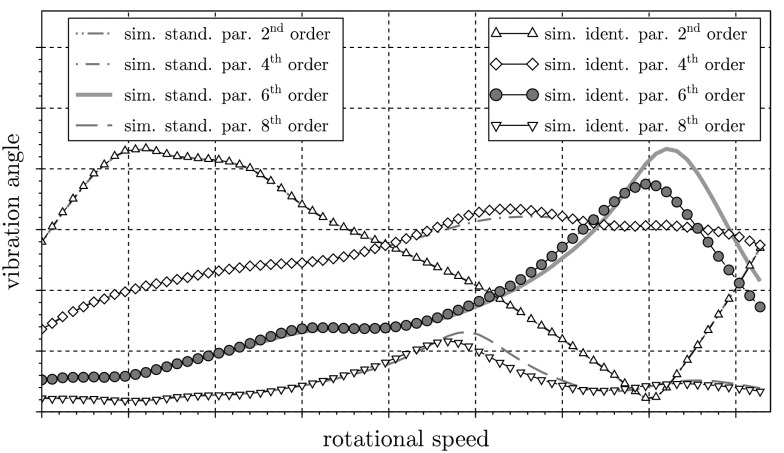
Comparison of the engine orders

In Fig. 13 the engine orders 2, 4, and 8 are depicted, too. On the one hand, the engine orders of the simulation with the initial parameters and, on the other hand, the engine orders of a simulation with the identified parameters of the TVD are shown. As expected, the spectra of the orders other than the 6th are only slightly affected.

## Conclusions

The present paper shows a new method for parameter identification using the amplitude response of oscillations in multibody system dynamics. The proposed method combines the classical Fourier analysis with the adjoint sensitivity analysis for the gradient computation in an optimization problem. Using the spectrum of a system output helps in understanding the behavior of a system whereas the interpretation of the time domain data is not always promising because of excessive time ranges, noisy signals, or systematic errors.

The method is applied to a nonlinear cart–pendulum system. In order to demonstrate that the method copes with system uncertainties in the example, one parameter, which is not part of the identification, is set to a value different from the one used for generating the measurement. Therefore, the desired spectrum cannot be obtained to its full extent, but only in the given frequency range. In the time domain this would lead to an optimization problem that is not well-posed.

Further, an identification is performed using the model of a four-cylinder engine. Here, the robustness of the method and also the capability to deal with larger systems are pointed out.

## Appendix A: The model equations

The equations of motion of the four-cylinder engine are summarized here. As mentioned in Sect. 4, the model equations are in the form

$$ \begin{aligned} \mathbf{M}(\mathbf{q}) \ddot{\mathbf{q}} + \mathbf{C}_{\mathbf {q}}^{\mathsf{T}}(\mathbf{q})\boldsymbol{\lambda}&= \mathbf{f}(\mathbf {q},\dot{\mathbf{q}},\mathbf{p},t), \\ \mathbf{C}(\mathbf{q}) &= \textbf{0}, \end{aligned} $$

where $\mathbf{q}$ is the vector of redundant degrees of freedom given by

$$ \mathbf{q}= [\underbrace{q_{1},q_{2},\dots,q_{6}}_{\text{crankshaft}}, \underbrace{q_{7},q_{8},\dots,q_{18}}_{\text{conrod}}, \underbrace {q_{19},q_{20},\dots,q_{22}}_{\text{piston}}, \underbrace {q_{23},q_{24},q_{25}}_{\text{TVD}}, \underbrace{q_{26}}_{\text{pulley wheel}} ]^{\mathsf{T}}. $$

Hence, the resulting global mass matrix is constant and reads

$$ \begin{aligned} \mathbf{M}= \text{diag}(&m_{1},m_{2},m_{3},m_{4},m_{5},m_{6},m_{cr},m_{cr},J_{cr}, \\ &m_{cr},m_{cr},J_{cr},m_{cr},m_{cr},J_{cr},m_{cr},m_{cr},J_{cr} \\ &m_{p},m_{p},m_{p},m_{p},J_{\text{TVD}},0,0,J_{\text{PW}}). \end{aligned} $$

Note that we have canceled the degree of freedom of the secondary side of the dual mass flywheel, because its motion is given by a function $q_{\text{runup}}(t)$. Hence, we exchange the degree of freedom of the dual mass flywheel $q_{23}$ by the given function $q_{\text{runup}}(t)$.

The corresponding force vector is

$$ \mathbf{f}= \begin{pmatrix} -F_{\text{DMF}}(\mathbf{q},q_{0}(t)) + d_{\text{DMF}} (\dot{q}_{0}(t) - v_{1}) + c_{1}(q_{2} - q_{1} - 2\pi) + d (v_{2} - v_{1}) \\ c_{1}(q_{1} - q_{2} + 2\pi) + d(v_{1} - v_{2}) + c_{2}(q_{3} - q_{2} + \pi) + d(v_{3} - v_{2}) \\ c_{2}(q_{2} - q_{3} + \pi) + d(v_{2} - v_{3}) + c_{3}(q_{4} - q_{3} - 2\pi) + d(v_{4} - v_{3}) \\ c_{3}(q_{3} - q_{4} - 2\pi) + d(v_{3} - v_{4}) + c_{4}(q_{5} - q_{4} + 3\pi) + d(v_{5} - v_{4}) \\ c_{4}(q_{4} - q_{5} + 3\pi) + d(v_{4} - v_{5}) + c_{5}(q_{6} - q_{5}) + d(v_{6} - v_{5}) \\ F_{6}(\mathbf{q},\dot{\mathbf{q}}) \\ 0\\0\\0\\0\\0\\0\\0\\0\\0\\0\\0\\0\\ -p_{1}(\mathbf{q},\dot{\mathbf{q}}) A_{P}\\ -p_{2}(\mathbf{q},\dot{\mathbf{q}}) A_{P}\\ -p_{3}(\mathbf{q},\dot{\mathbf{q}}) A_{P}\\ -p_{4}(\mathbf{q},\dot{\mathbf{q}}) A_{P}\\ c^{*}_{\text{par}}(q_{6} - q_{23}) + c^{*}_{1}(q_{24} - q_{23}) + c^{*}_{2}(q_{25} - q_{23}) \\ c^{*}_{1}(q_{23} - q_{24}) + d^{*}_{1}(v_{24} - v_{6})\\ c^{*}_{2}(q_{23} - q_{25}) + d^{*}_{2}(v_{25} - v_{6})\\ c_{\text{PW}}(q_{6} - q_{26}) + d_{\text{PW}}(v_{6} - v_{26}) \end{pmatrix} , $$

where the $6{\text{th}}$ entry is

$$ \begin{aligned} F_{6}(\mathbf{q},\dot{\mathbf{q}}) &= c_{5}(q_{5} - q_{6}) + d(v_{5} - v_{6}) + c^{*}_{\text{par}}(q_{23}-q_{6}) \\ &\quad{}+ d^{*}_{1}(v_{6} - v_{24}) +d^{*}_{2}(v_{6} - v_{25}) \\ &\quad{}+ c_{\text{PW}}(q_{26} - q_{6}) + d_{\text{PW}}(v_{26} - v_{6}). \end{aligned} $$

The first term in the force vector $F_{\text{DMF}}(\mathbf {q},q_{0}(t))$ is given by a nonlinear stiffness characteristic.

The crankshaft, conrods, and the pistons are described with redundant coordinates. Hence, we introduce four algebraic constraint equations for each cylinder, where $r_{cs}$ is the radius of the crankshaft, $l_{cr}$ is the length of the conrod, and $s_{cr}$ is the distance to the center of mass.

Finally, the vector of constraint equations $\mathbf{C}(\mathbf{q})$ is given by

$$ \begin{aligned} \mathbf{C}(\mathbf{q}) = \begin{pmatrix} r_{cs} \sin(q_{2}) - l_{cr} \sin(q_{9})\\ q_{7} + r_{cs} \sin(q_{2}) - s_{cr}\sin(q_{9})\\ q_{8} - r_{cs} \cos(q_{2}) - s_{cr}\cos(q_{9})\\ q_{19} - r_{cs} \cos(q_{2}) - l_{cr}\cos(q_{9})\\ r_{cs} \sin(q_{3}) - l_{cr} \sin(q_{12})\\ q_{10} + r_{cs} \sin(q_{3}) - s_{cr}\sin(q_{12})\\ q_{11} - r_{cs} \cos(q_{3}) - s_{cr}\cos(q_{12})\\ q_{20} - r_{cs} \cos(q_{3}) - l_{cr}\cos(q_{12})\\ r_{cs} \sin(q_{4}) - l_{cr} \sin(q_{15})\\ q_{13} + r_{cs} \sin(q_{4}) - s_{cr}\sin(q_{15})\\ q_{14} - r_{cs} \cos(q_{4}) - s_{cr}\cos(q_{15})\\ q_{21} - r_{cs} \cos(q_{4}) - l_{cr}\cos(q_{15})\\ r_{cs} \sin(q_{5}) - l_{cr} \sin(q_{18})\\ q_{16} + r_{cs} \sin(q_{5}) - s_{cr}\sin(q_{18})\\ q_{17} - r_{cs} \cos(q_{5}) - s_{cr}\cos(q_{18})\\ q_{22} - r_{cs} \cos(q_{5}) - l_{cr}\cos(q_{18})\\ \end{pmatrix} = \textbf{0}. \end{aligned} $$

From Fig. 8 the constraint equations can be assembled. The first constraint equation describes the $x$-component of the big end bearing. The second and third constraint equations link the center of mass of the conrod with the redundant generalized coordinates, while the fourth constraint equation describes the piston’s center of mass.

## Appendix B: The Hanning Window

Let us consider a signal of the form

$$ f(t) = \frac{a_{0}}{2} + \sum_{k=1}^{N} \bigl[ a_{k} \cos(\omega_{k} t) + b_{k} \sin( \omega_{k} t) \bigr] $$

with $\omega_{k} = k \omega= k \frac{2\pi}{T}$ and the period duration $T$. Using the Hanning window function

$$ h(t) = \frac{1}{2} \biggl[ 1-\cos \biggl( \frac{2\pi}{n T} \;t \biggr) \biggr] , $$

the Fourier-coefficient of the $i{\text{th}}$ engine order ($i \geq1$) can be computed by

$$ \bar{a}_{i} = \frac{2}{n\,T} \int_{0}^{n\,T} f(t) h(t) \cos(i\,\omega \,t)\, \mathrm{d}t $$

where $n$ is the number of periods to be considered. For $n=1$ the Fourier coefficient $\bar{a}_{i}$ is

$$ \bar{a}_{i} = \lim_{n \rightarrow1}{\frac{2}{n\,T} \int _{0}^{n\,T} f(t) h(t) \cos(i\,\omega\,t) \,\mathrm{d}t} = \frac {1}{4} ( -a_{i-1} + 2 a_{i} - a_{i+1} ) $$

and for $n>1$ the Fourier coefficient $\bar{a}_{i}$ is

$$ \bar{a}_{i} = \frac{2}{n\,T} \int_{0}^{n\,T} f(t) h(t) \cos(i\,\omega \,t)\,\mathrm{d}t= \frac{1}{2} a_{i}. $$

It has been shown that more than one period is required when using a Hanning window. Otherwise, the amplitude $\bar{a}_{i}$ includes information from other harmonics, which leads to wrong results. Furthermore, the amplitude $\bar{a}_{i}$ has to be corrected by a factor of 2 to be comparable with measured amplitudes.
